# Supplementary material for: How Uncertainty Affects Children's Exploration and Exploitation in Statistical Learning
Source: Dev Sci. 2026 Jun 11;29(4):e70235. doi: 10.1111/desc.70235 (PMC13260883; doi:10.1111/desc.70235)
Supplement: Supplementary file 1 — Supporting Information: desc70235‐sup‐0001‐figuresS1‐S4.docx [file DESC-29-e70235-s001.docx]

*Figure S1*

Age Effect on First Fixation Position


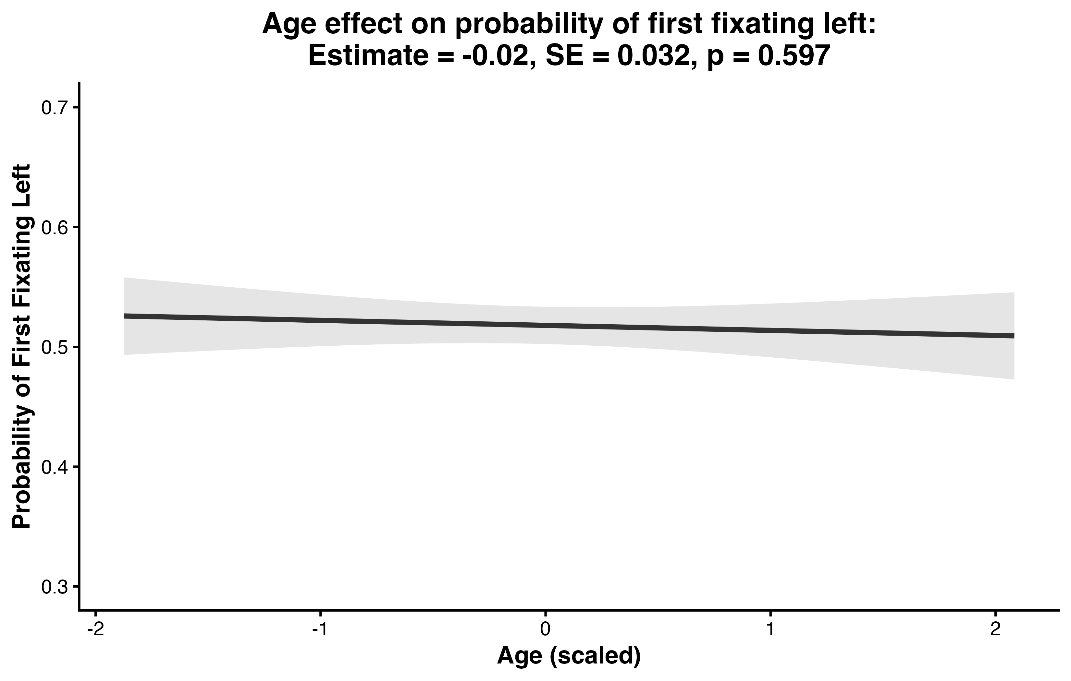


*Figure S2*

Effects of Age and Probe Position on First Fixation Latency


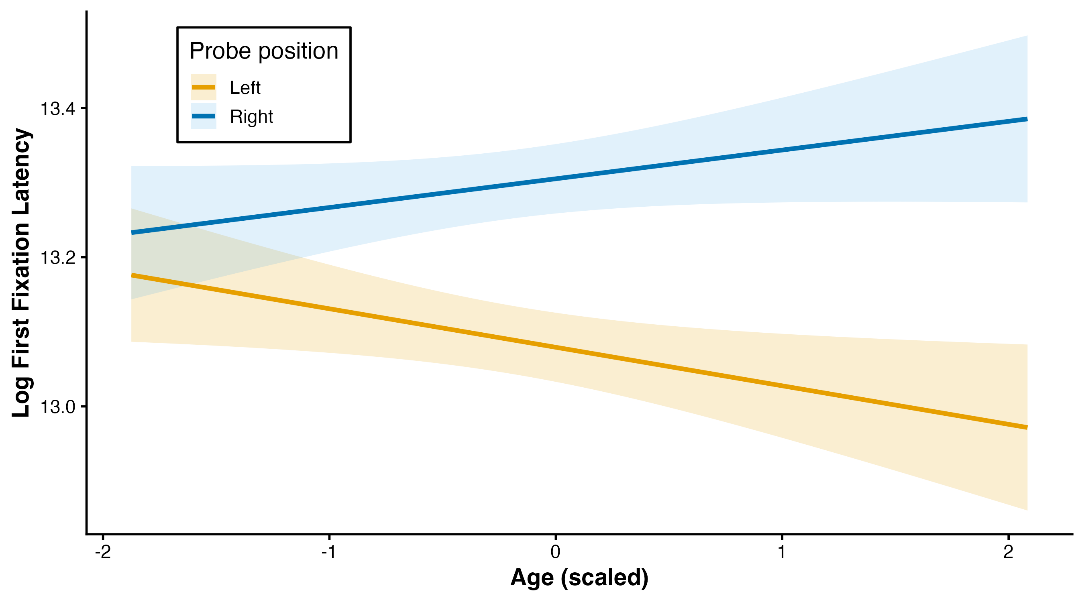


*Figure S3*

The posterior prediction checks for the total duration model.

*
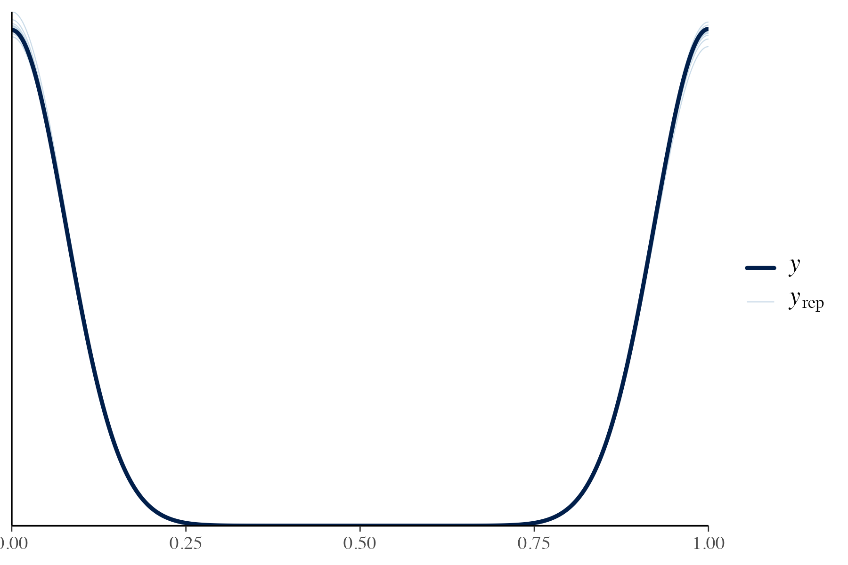
*

*Note.* The plot of model-simulated density (light blue lines) in ten runs and the density from the real data (dark blue line).

*Figure S4*

The posterior prediction check for the first fixation duration model.

**
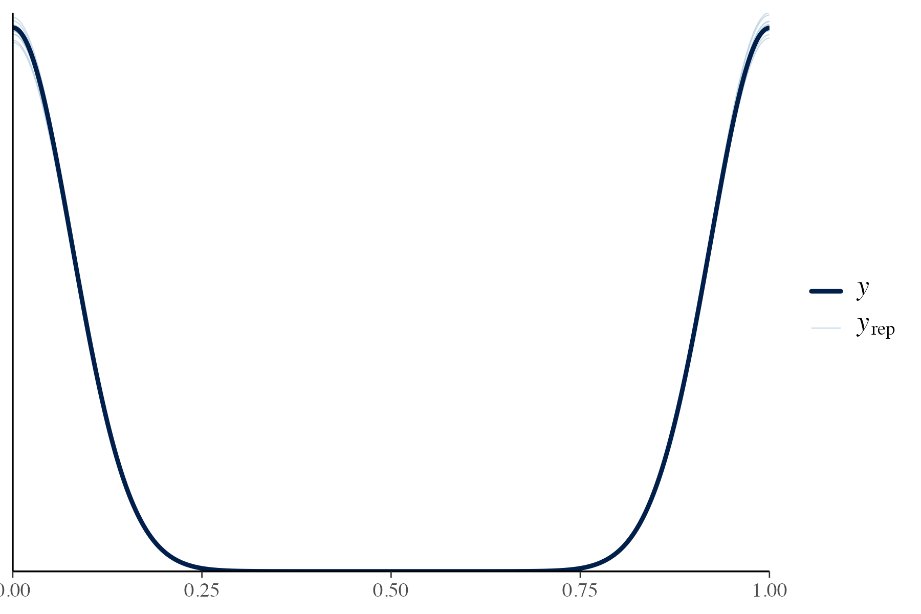
**

*Note.* The plot of model-simulated density (light blue lines) in ten runs and the density from the real data (dark blue line).
